# Correction: MicroRNA-21 promotes TGF-β1-induced epithelial-mesenchymal transition in gastric cancer through up-regulating PTEN expression

**DOI:** 10.18632/oncotarget.27729

**Published:** 2020-08-25

**Authors:** Chang Li, Lei Song, Zhuo Zhang, Xiao-Xue Bai, Ming-Fu Cui, Lian-Jun Ma

**Affiliations:** ^1^ Department of Gastrointestinal Surgery, China-Japan Union Hospital of Jilin University, Changchun, 130000, Jilin, P.R. China; ^2^ Department of Respiratory Medicine, The First Hospital of Jilin University, Changchun, 130000, Jilin, P.R. China; ^3^ Department of Orthopaedics, China-Japan Union Hospital of Jilin University, Changchun, 130000, Jilin, P.R. China; ^4^ Department of Cadre Ward, The First Hospital of Jilin University, Changchun, 130000, Jilin, P.R. China; ^5^ Department of Endoscopics, China-Japan Union Hospital of Jilin University, Changchun, 130000, Jilin, P.R. China

**This article has been corrected:** Due to errors during typesetting, the image for Figure 4D displays the wrong group. The corrected Figure 4 is shown below. The authors declare that these corrections do not change the results or conclusions of this paper.

Original article: Oncotarget. 2016; 7:66989–67003. 66989-67003. https://doi.org/10.18632/oncotarget.11888

**Figure 4:**
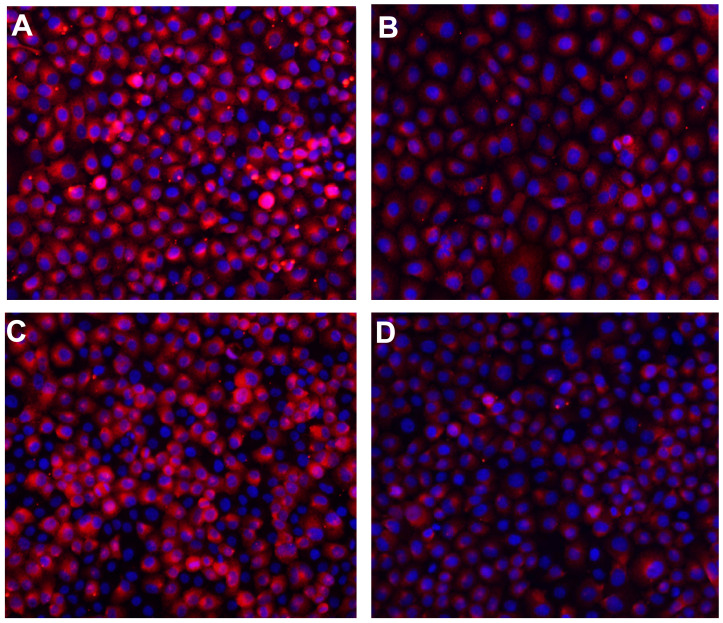
Immunofluorescence staining of E-cadherin expression in SGC-7901 and KATO-III cells after TGF-β1 treatment for 48 h (×200). (**A**) E-cadherin expression in SGC-7901 cells in the BSA control group; (**B**) E-cadherin expression in SGC-7901 cells with TGF-β1 treatment; (**C**) E-cadherin expression in KATO-III cells in the BSA control group; (**D**) E-cadherin expression in KATO-III cells with TGF-β1 treatment). Note: TGF-β1, transforming growth factor β1; GC, gastric cancer; BSA, bovine serum albumin.

